# Unifying Divergent Conceptions in Nonfluent/Agrammatic and Semantic Primary Progressive Aphasia

**DOI:** 10.3390/brainsci16050509

**Published:** 2026-05-09

**Authors:** Marc Teichmann, Kimihiro Nakamura

**Affiliations:** 1 French Reference Center for ‘Rare or Early Onset Dementias’, Institute of Memory and Alzheimer’s Disease, Department of Neurology, Pitié-Salpêtrière University Hospital, AP-HP, 47-83 boulevard de l’Hôpital, 75013 Paris, France; 2 Section of Systems Neuroscience, National Rehabilitation Center for Persons with Disabilities, Tokorozawa 359-8555, Japan

**Keywords:** primary progressive aphasia, primary progressive apraxia of speech, language

## Abstract

**Highlights:**

1. There is no consensus whether nonfluent/agrammatic PPA should include primary progressive apraxia of speech (AOS) (lumping/continuum view) or not (splitting view), and whether semantic PPA should be dissociated from the ‘right temporal variant of semantic dementia’ (splitting), or not (continuum).
2. Combining findings from neurocognitive, neurobiological and clinical research helps to overcome ‘lumping’ vs. ‘splitting’ controversies by supporting the conception of ‘graded continua’ with phenotypical subdivisions: syntactic/phonological/phonetic-AOS phenotypes within the language/speech production continuum (Broca-premotor/SMA network), verbal/non-verbal/behavioral phenotypes within the multidimensional semantic space (anterior temporal lobe network).
3. Adopting such integrative-graded conceptions might provide comprehensive frameworks guiding clinical reasoning, care strategies and research in the two proposed continua/spectrum diseases and their phenotypical subdivision variants.

**Abstract:**

The nonfluent/agrammatic variant of primary progressive aphasia (nfav-PPA) and primary progressive apraxia of speech (PPAOS) are neurodegenerative syndromes that raise diagnostic challenges related to several issues. First, there are two divergent conceptions, one stipulating that (i) nfav-APP and PPAOS are distinct entities, and the other (ii) that PPAOS has to be integrated into the nfav-APP spectrum. A second related issue concerns the consideration of phonological dimensions, lying at the language interface with speech, which could potentially help overcome the nfva-PPA/PPAOS controversy. Third, there is a lack of internationally validated clinical tests assessing apraxia of speech and syntactic abilities with sufficient specificity and sensitivity. This narrative review discusses these issues taking into account clinical, neurocognitive and neurobiological dimensions. It proposes a conceptual-integrative framework conciliating competing nfav-APP/PPAOS accounts while suggesting a graded continuum with subdivisions, related to neurodegenerative expansion throughout language/speech production systems, ranging from syntactic to phonological to phonetic-articulatory impairments. A second controversy in the field of PPA arises from divergent conceptions of semantic PPA (sv-PPA), defined by primary damage to verbal semantics, and of semantic dementia (SD) characterized by multimodal semantic impairments. The current consensus criteria of PPA have deconstructed the initial SD conception by absorbing it into sv-PPA, hence leaving mixed and some non-verbal semantic phenotypes nosologically orphaned. Again, the article proposes a conceptual and integrative model, built on findings from clinical research and cognitive neuroscience, suggesting a graded continuum with subdivisions spanning from verbal to different non-verbal semantic impairments including social-semantic/behavioral phenotypes.

## 1. Introduction

**Figure 1:**
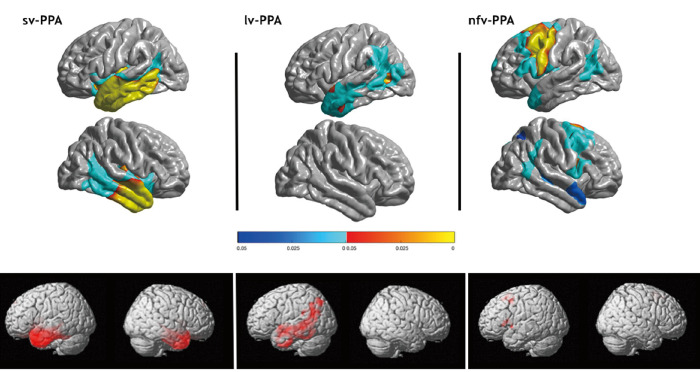
Cortical regions damaged in the three canonical PPA variants: sv-PPA, lv-PPA, nfv-PPA = nfav-PPA (adapted from Routier et al., 2018) [8]. Top panel: Areas of significantly reduced cortical thickness on MRI compared to healthy controls. Corrected *p*-values at the vertex level and the cluster level are displayed with red/yellow and blue colors, respectively. Bottom panel: Areas of significant hypometabolism on FDG-PET compared to healthy controls. Maps display *p*-values, corrected for multiple comparisons using peak-level FWE correction (*p* < 0.05).

Primary progressive aphasias (PPAs) represent a group of neurodegenerative syndromes mainly affecting language processing. The current international consensus criteria for diagnosis of the three canonical variants (nonfluent/agrammatic [nfav-PPA], semantic [sv-PPA], logopenic [lv-PPA]) are based on clinical core features and on specific imaging patterns regardless of the underlying proteinopathy [1] (see Figure 1). Up to 30–40% of PPA patients are not captured by this tripartite classification, which generated the by-default label of ‘mixed’ or ‘unclassifiable’ PPA [2,3,4]. Such PPA presentations include ‘incomplete’ phenotypes such as pure progressive agrammatic aphasia [5] and mixtures of language disorders, reflecting progressive spread of neurodegeneration. There is, however, a critical issue regarding pure progressive apraxia of speech (AOS) manifesting as articulation disorders but satisfying current criteria of nfav-PPA. AOS has been defined as deficient programming of articulatory gestures [6], leading primarily to phonetic impairments (phonemic distortions), and to prosodic deficits. Yet, phonetic impairments arise downstream of language processing, following phonological encoding, which has encouraged several authors to individualize ‘primary progressive apraxia of speech’ (PPAOS) and to exclude the entity from the PPA spectrum [7]. The AOS issue furthermore tackles the challenge to delineate phonetic speech programming errors from motor-related dysarthria. Clinicians face such competing PPAOS/nfav-PPA conceptions, which has implications for diagnosis, patient care, and potentially for prognosis and prediction of underlying proteinopathies.

A second controversy within PPA is linked to the conceptual and terminological rivalry between ‘semantic PPA’ (sv-PPA) and ‘semantic dementia’ (SD), which might extend towards ‘right temporal forms of SD’. The current consensus criteria for PPA diagnosis [1] have deconstructed the initial concept of SD, defined by multimodal semantic deficits [9], while absorbing it into the PPA spectrum. Accordingly, sv-PPA is characterized by primary breakdown of verbal semantics, and only incidentally by impairments of non-verbal object semantics. However, the concept of the multimodal SD phenotype appears to remain appropriate to designate progressively emerging stages of semantic impairment reflecting neurodegenerative expansion within the semantic network. Hence, ‘sv-PPA’ and ‘SD’ phenotypes might be part of a time-constrained graduated continuum, which may also encompass phenotypes with primary impairment of non-verbal semantics. Such phenotypes include pure/predominant damage to visual object semantics and presentations with primary disorders of social/emotional semantics. The former phenotype currently lacks a formal nosological label, while the latter is integrated into the entity of ‘right temporal variant of frontotemporal dementia (FTD)’ [10] or ‘semantic-behavioral variant FTD’ [11], characterized by predominant social/emotional-behavioral disorders.

Taken together, there are two distinct axes of controversy in the current nosology of PPA, one related to language/speech production and the other to semantic processing. For each of them, we will propose unifying conceptual frameworks of ‘graded continua’ related to progressive neurodegenerative expansion, spanning (i) from pure/predominant AOS to phonological impairments to agrammatism, and (ii) from verbal to different non-verbal semantic disorders.

Relevant literature for this narrative review suggesting an integrative modelling approach was screened using Pubmed. The search was performed in January 2026 using a list of keywords, which included ‘primary progressive aphasia’, ‘semantic primary progressive aphasia’, ‘semantic dementia’, ‘apraxia of speech’, ‘frontotemporal dementia’, ‘semantic’, ‘syntax’, and their combination using Boolean operators (‘AND’, ‘OR’).

## 2. nfav-PPA and PPAOS

### 2.1. Speech and Language

According to the consensus criteria, nfav-PPA is characterized by progressive agrammatism or AOS [1]. Agrammatism is caused by damage to a core network of language processing underpinning combinatorial computations of syntax. Conversely, AOS characterized by speech-sound distortions is related to impaired phonetic encoding occurring after phonological language computations [12]. This basic distinction between speech/articulation programming and language processes has encouraged the separation of PPAOS from PPA [7]. Such a distinction is also consistent with anatomical/imaging findings of neurolinguistic research showing that combinatorial syntax is implemented by posterior-inferior prefrontal cortices (‘Broca’s region’), whereas phonetic encoding primarily involves premotor cortices including the supplementary motor area (SMA) [12,13,14,15] as well as opercular-insular regions [16,17]. Coherently, imaging studies in ‘pure’ PPAOS reveal damage to premotor cortices involving the SMA [7,18], and imaging in nfav-PPA series with patients having agrammatism and/or AOS show damage to both Broca’s region and adjacent premotor cortices [8,19] (see Figure 1). In brief, findings of language and neurolinguistic research provide concordant evidence for an anatomo-functional distinction between phonetic encoding (AOS) and syntax (agrammatism), involving distinct computational mechanisms and topographically close but distinct cortical regions. Such evidence appears to legitimate the separation between PPAOS and nfav-PPA. However, expansive neurodegeneration is known to start in local and specialized neural systems, subsequently spreading to other brain regions with strong structural and functional connectivity [20,21]. Expanding neuronal degeneration is therefore likely to produce merged phenotypes within a graded continuum ranging from pure/predominant AOS to pure/predominant agrammatism, while encompassing mixed presentations.

### 2.2. nfav-PPA and Phonology

There is yet another issue regarding the current definition of nfav-PPA. According to findings of cognitive neuroscience, Broca’s region, damaged in nfav-PPA, incorporates the neural equipment for computations of combinatorial language processes, which are relevant for both syntactic compositionality and phonological concatenation [12,15,22,23]. It is therefore intriguing that consensus criteria did not integrate phonological disorders of phoneme combining into the core criteria for nfav-PPA diagnosis. Instead, phonological paraphasias are part of the diagnosis criteria of the logopenic variant of PPA (lv-PPA), which is characterized by predominant damage to the temporal-parietal junction [1] implementing phonological word representations and phonological short-time memory [12,24]. This situation might be explained by the fact that consensus criteria considered phoneme inversions/substitutions/omissions, resulting from failure of phonological encoding, to be part of AOS. The chapter defining nfav-PPA stipulates that ‘patients typically make inconsistent speech sound errors, consisting of distortions, deletions, substitutions, insertions, or transpositions of speech sounds’. Such a position however reveals discrepancies between clinical criteria and cognitive neuroscience linking Broca’s region to phonological encoding, and inconsistencies with respect to the definition of AOS considered to be a praxis and not a language disorder [6]. Several studies have tackled this issue, indicating that phonological errors leading to phonemic paraphasias appear to be more frequent in nfav-PPA than phonetic shifting [25], and that phonological encoding as such is altered in nfav-PPA but not in lv-PPA [26]. Furthermore, phonological paraphasias have been shown to be more frequent in nfav-PPA than in lv-PPA [27,28,29], and phonological paraphasias in lv-PPA have been shown to be less frequent than semantic paraphasias [28,30]. Taken together, in addition to phonetic/AOS disorders, phonological encoding impairments might be taken into account in patients with nfav-PPA, which may lead to the individualization of predominant phonological phenotypes.

### 2.3. Biological Aspects

Neuropathological studies have shown that both nfav-PPA and PPAOS are mainly caused by underlying 4R-tauopathies such as corticobasal degeneration (CBD, 30–55%), progressive supranuclear palsy (PSP, 20–30%) or Pick’s tauopathy (5–20%). In some cases TDP-43 proteinopathies can be causative (5–25%), but underlying Alzheimer pathology appears to be rare (<5%) [3,31,32,33]. Comparing two of the largest neuropathological series on nfav-PPA (*n* = 109) [33] and PPAOS (*n* = 32) [34] suggests that underlying CBD is more prevalent than PSP in nfav-PPA (29% and 17%, respectively) and in PPAOS (53% and 31%, respectively), and that TDP-43 proteinopathy is rarer in PPAOS (6%) than in nfav-PPA (24%). One study reporting a direct comparison between nfav-PPA (*n* = 33), PPAOS (*n* = 3) and pure agrammatic aphasia (*n* = 4) also suggested that underlying CBD is more frequent in the nfav-PPA group (42%) than PSP pathology (18%) [35]. However, there are no investigations in large patient samples contrasting and matching the different tauopathies and TDP-43 proteinopathies with the different clinical phenotypes of a graded nfav-PPA/PPAOS spectrum including syntactic/agrammatic, phonetic/AOS, and possibly phonological variants.

### 2.4. Clinical Aspects

One critical issue for clinical research is that studies on nfav-PPA might be submitted to biases related to lumping together AOS, agrammatic and phonological phenotypes, which can lead to non-homogeneous patient cohorts. Second, there is a lack of clinical tests detecting and quantifying AOS with sufficient sensitivity and specificity while distinguishing it from dysarthria. The few existing standard tests are examiner-dependent and submitted to subjective biases (e.g., ‘Apraxia of Speech Rating Scale’, ‘Motor Speech Evaluation’ [36,37]). This situation generates diagnostic uncertainties, which can lead to erroneous inclusions of patients with progressive dysarthria into nfav-PPA or PPAOS cohorts, or even to being confounded with motor system diseases such as bulbar forms of amyotrophic lateral sclerosis. On the other hand, progress of AOS detection has been made using automated measures of speech timing including articulatory rate (number of syllables per time unit) and of intra-word vowel/syllable durations. The latter parameter operationalized as the ‘pairwise variability index’ appears to be more specific to AOS than to other motor speech disorders, and articulatory rates correlate with cortical thickness of premotor regions including the SMA [38,39]. Such experimental findings might form a basis for the development of clinical tests adapting speech timing parameters, which need to be validated with patients with AOS and with different types of dysarthria. In the meantime, proxy markers of AOS should aid in diagnosis, such as clinical detection of orofacial apraxia, which is related to AOS in about 70% of patients with nfav-PPA [40]. In addition, FDG-PET can sensitively reveal hypometabolism of premotor/AMS and/or opercular-insular regions, which have been shown to be damaged in patients with pure/predominant AOS [7,16,18]. Finally, difficulties in distinguishing AOS from motor-related dysarthria could encourage clinicians to perform electromyography, detecting signs of amyotrophic lateral sclerosis, and to look for extrapyramidal motor symptoms given the frequent evolution of nfav-PPA towards corticobasal or PSP syndromes.

In addition to the lack of standard tests for AOS, there is also a shortage of clinical tests reliably detecting and quantifying syntactic disorders with sufficient sensitivity and specificity. In particular, currently available tests can hardly disentangle syntactic failure from other factors influencing sentence processing such as short-term memory or executive functioning. Such biases also apply to tests specifically developed for quantifying syntactic disorders in PPA, like the ‘Northwestern Anagram Test’, involving a cognitively demanding procedure including the manipulation of printed words, organizing them into sentences while trying to match sentences with pictures [41].

Taken together, in the absence of clinical tests providing objective/reproducible and quantitative measures of AOS and syntactic failure, the gold standard of nfav-PPA diagnosis remains ‘expert opinion’, which can lead to diagnostic errors and selection/inclusion biases, especially in retrospective PPA investigations.

## 3. sv-PPA and SD

### 3.1. Nosology

The current PPA consensus criteria [1] define sv-PPA by isolated or largely predominant impairment of verbal semantics (naming, single-word comprehension) related to predominant damage to the left anterior temporal lobe (ATL), whereas previous criteria of SD require multimodal, verbal and non-verbal, semantic impairments [9]. The controversy between SD and sv-PPA is driven by historical, terminological and conceptual factors. PPA, including a fluent/semantic variant, has initially been individualized by Marsel Mesulam [42], whereas SD characterized by multimodal semantic impairments has been described by another line of clinical research [9,43,44]. Mesulam et al. [45] have claimed that semantic PPA is caused by the disruption of the left-sided language network affecting specifically the left ATL, whereas syndromes with impaired object recognition would involve distinct networks. Conversely, several authors have posited that semantic PPA and SD belong to the same entity caused by progressive damage to a multimodal semantic system, bilaterally distributed in the left and right ATL [46]. Accordingly, semantic aphasia was proposed to be an early-stage presentation of SD. The current international PPA consensus criteria [1] have abandoned the SD entity defining sv-PPA as a language disease affecting exclusively or predominantly verbal semantics due to left ATL damage. On the other hand, a novel syndromic entity related to predominant right ATL damage has been individualized, labeled ‘right temporal variant FTD’ or ‘semantic-behavioral variant FTD’ [10,11], presenting with disorders of non-verbal/visual and social/emotional semantics resulting in predominant behavioral disturbances.

### 3.2. The ‘Semantic Hub’ of the Anterior Temporal Lobes

This historical evolution of clinical considerations points to the fundamental issue of whether there is a unique supra-modal semantic system (‘semantic hub’) bilaterally distributed in the ATLs (unitary ‘hub-and-spoke’ model) [47], or whether there are different modality-specific systems including a left ATL network dedicated to verbal semantics (hemispheric specialization model) [48,49,50]. The hemispheric specialization account has primarily been supported by findings with SD patients having predominant left- or right-sided damage, suggesting that verbal and non-verbal semantics might depend on the left and right ATL, respectively [51,52,53]. However, pure unilateral ATL damage occurs only in the initial stages of neurodegeneration, and clear functional left/right ATL distinctions could not be formally established. Likewise, imaging/behavior studies in patients using correlation analyses are unable to infer causal relationships. Basic neuroscience research in healthy subjects has used fMRI [54] and TMS [55], indicating bilateral ATL involvement for both verbal and non-verbal semantics, thus favoring a supra-modal semantic hub redundantly distributed in both ATLs. Subsequent imaging studies have indicated some degree of functional specialization within the ATL system [56], leading to the concept of a graded semantic hub [47]. Further specification has been provided by TMS, inferring causality, coupled with priming tasks taping implicit/automatic semantic processing, thus avoiding biases related to non-semantic/executive factors. This methodological approach has unveiled that verbal semantics exclusively depends on left ATL portions whereas visual object semantics depends on the right ATL and on additional verbal feedback delivered by the left ATL [57]. Regarding more specifically non-verbal semantics, studies on sensory modalities other than the visual input/output channels are rare, but several studies have investigated semantic processing related to ‘abstract’ representations such as socially or emotionally relevant concepts [58,59,60,61]. Some of these studies looking at left/right lateralization point to a predominant role of the right ATL in social semantics and in ‘theory of mind’ processing, which shape elementary aspects of human behavior [58,62,63,64].

Altogether, semantic modelling based on cognitive neuroscience provides a foundation for understanding clinical phenotypes caused by expanding neurodegeneration within the ATL system, comprehending sv-PPA (predominant impairment of verbal semantics), SD (multimodal/mixed semantic impairment), and ‘right temporal forms of SD’/‘right temporal variant FTD’ (non-verbal/visual and behavioral impairments). In other words, such a graded phenotypical continuum with functional subdivisions points to a disease spectrum spanning from verbal to non-verbal/visual to social/behavioral variants of ‘semantic degeneration’ with initially distinct but progressively overlapping clinical presentations.

### 3.3. Biological Aspects

Neuropathological studies have shown that sv-PPA/SD are mainly caused by underlying TDP-43 type C proteinopathy, while causative tauopathies are rarer, and Alzheimer pathology seems to be rather exceptional. One of the largest neuropathological series including 106 cases [33] showed that 80% were related to underlying TDP-43 pathology, 11% to tauopathies and 5% to Alzheimer lesions. A comparative study investigating TDP-43 type C pathology in predominant left ATL vs. right ATL cases (*n* = 30) reported that TDP-43 pathology equally affects the left ATL (60% of the cases) and the right ATL (40%) [65]. However, there are no investigations in large patient samples specifically contrasting neuropathological patterns (TDP-43, tauopathies, Alzheimer) and the different clinical phenotypes of the semantic degeneration spectrum.

## 4. Unified but Graded Frameworks for ‘nfav-PPA–PPAOS’ and ‘sv-PPA–SD’

A major issue for neurology is to conciliate findings of basic neuroscience with clinical research and practice to ensure overall consistency. Regarding language/speech production and semantics, cognitive neuroscience tells a coherent story, which is key for overcoming the neurological ‘nfav-PPA/PPAOS’ and ‘sv-PPA/SD/right temporal FTD’ controversies, suggesting a unifying view of ‘graded continua’ (spectrum diseases) incorporating phenotypical subdivisions (variants). Such a view is neutral regarding underlying biological processes, but it tackles the question whether subdivision variants of the continua might be differentially related to tauopathies, TDP-43 proteinopathies or Alzheimer pathology. The conceptual framework of graded continua also builds on previous articles discussing and questioning the unresolved issue opposing phenotypical ‘splitting’ and ‘lumping’ accounts [66]. Splitting PPAOS from nfav-PPA [7] versus lumping them together [1,35] and individualizing a ‘semantic-behavioral FTD’ variant [11] distinct from sv-PPA versus lumping them into the umbrella concept of ‘semantic dementia’ [67] has created conflicting views, heterogeneous clinical approaches and divergent terminologies. This unstable situation in clinical practice has indicated the need for unifying conceptualizations incorporating both continua, based on evidence from neuroscience, and phenotypical graduations, which are key to clinical care and research.

### 4.1. The nfav-PPA–PPAOS Debate

**Figure 2:**
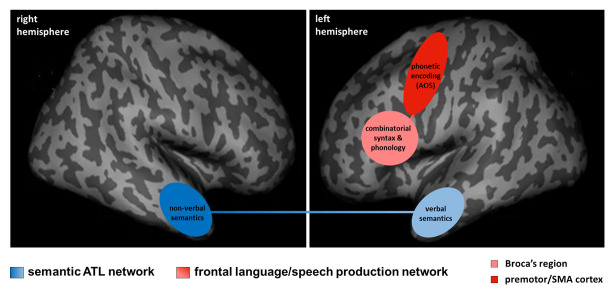
Illustration of the two brain networks implementing the graded continua involved in language/speech production and in verbal/non-verbal semantic processing.

With respect to the ‘nfav-PPA/PPAOS’ controversy, cognitive neuroscience has shown that parts of the posterior-inferior prefrontal cortex (‘Broca’s region’) and premotor-SMA regions are causally involved in combinatorial processing of syntax and phonology and in phonetic encoding, respectively. Consequently, damage to Broca’s region generates syntactic impairments (agrammatism) and phonological failure (phonemic paraphasias), and damage to the premotor-SMA regions results in phonetic breakdown (AOS). Imaging studies have demonstrated that Broca’s region and frontal premotor-SMA cortices are structurally and functionally connected by the frontal aslant tract [68,69,70], thus forming a neural core system for language/speech production (see Figure 2). Erosion of this system due to neurodegeneration, mediated by axonal degeneration of short inter-cortical connections [68,71], necessarily leads to progressive dysfunction of syntactic-phonological-phonetic processes with a time trajectory depending on the neurodegenerative start-point (AOS followed by phonological and syntactic breakdown, or the reverse). Such expanding structural damage throughout the Broca-premotor/SMA language/speech production network has been shown to occur in both nfav-PPA and PPAOS populations [38,72,73]. In clinical practice, after some years of disease evolution most patients have mixed but differentially weighted phenotypes, lying within a graded phenotypical continuum containing subdivisions corresponding to pure/predominant syntactic (‘progressive agrammatic aphasia’), phonological, or phonetic disorders (‘PPAOS’). The continuum concept is also coherent with clinical research investigations including, among others, a pivotal retrospective study with clinical and neuropathological data of patients with ‘progressive agrammatic aphasia’ (*n* = 6), ‘progressive AOS’ (*n* = 12) and a combination of both (‘nfav-PPA’, *n* = 75) [35]. Analyses on clinical data revealed the absence of clearly separable and temporally stable functional clusters, indicating that the different phenotypes could be integrated into a covering spectrum disease/syndrome due to 4R-taupathies. However, the absence of clear-cut statistical clusters, based on clinical tests, and small sample sizes do not preclude the absence of relevant graduations within the continuum. Taken together, ‘lumping’ (continuum) as opposed to phenotypical ‘splitting’ (subdivisions) views, which fuel a seemingly unsolved debate in the field of PPA [66], are not mutually exclusive but can combine into a clinically relevant ‘graded continuum’ account.

A critical issue, which is key to pharmacological therapy approaches, is the potentially differential vulnerability of Broca’s region (syntax/phonological first cases) and premotor-SMA regions (AOS first cases) to different proteinopathies. One of the largest neuropathological datasets [33] has shown that most nfav-PPA cases (including AOS and agrammatic subtypes) are related to underlying 4R-tauopathies (CBD, PSP), but the investigation has also demonstrated that TDP-43 proteinopathies can be causally involved. Studies centered on nfav-PPA or PPAOS suggest that both entities are more frequently related to CBD than to PSP pathology, which is compatible with an investigation contrasting PPAOS/agrammatic/nfav-PPA phenotypes while revealing that 14/33 patients of the nfav-PPA group had CBD vs. 6/33 with PSP [35]. However, evidence for the presence or absence of neurobiological subdivisions/vulnerabilities within a graded continuum of the Broca-premotor/SMA network is weak given the lack of largescale neuropathological investigations with homogenous patient populations having ‘pure’ AOS or agrammatism. Furthermore, phonological dimensions are not taken into account in patients with nfav-PPA, raising the question of whether they could contribute to indicate causative proteinopathies. In this context, the development of in vivo markers of 4R-taupathies and TDP-43 proteinopathies will be crucial for elucidating whether distinct clinical presentations, such as AOS/agrammatic/phonological predominant phenotypes, might match and predict specific or prevalent neurobiological entities.

A second issue of the continuum view, which is of critical clinical relevance, is the incorporation of phenotypical phonetic/phonological/syntactic subdivisions that would be key for appropriate clinical reasoning and care, including targeted speech therapy approaches. Likewise, anatomical subdivisions within a graded continuum might be essential for non-pharmacological approaches interacting with brain structures, such as non-invasive brain stimulation. A last issue could potentially be to develop diagnosis criteria of the proposed tripartite language/speech disorder spectrum, including specific characterizations of its phonetic/AOS, phonological and agrammatic phenotypes, while possibly agreeing on formal label names. Such an approach could be helpful for clinical reasoning and for providing a consistently structured basis for clinical research. In this respect, some nosological label names have already been proposed, including ‘progressive non-fluent speech and aphasia spectrum’ [17], which has the merit to take into account the existence of speech/language distinctions within a syndrome continuum. Importantly, labels might account for phenotypical subdivisions, which could include predominant phonetic variants (‘PPAOS’ [7]), syntactic variants (‘primary progressive agrammatism’ [5]), and phonological phenotypes that currently lack a formal name.

### 4.2. The sv-PPA–SD Debate

Neuroscience has generated two key models of semantics: the ‘unitary supramodal hub-and-spoke model’ [47] and the ‘hemispheric specialization model’ [50]. Despite their differences, both attribute a central role to the bilateral ATL system. Both are globally reconcilable given that they posit segregation of the system, which is binary (left ATL/verbal vs. right ATL/non-verbal) in the hemispheric specialization model and more graded in the supra-modal account. Both models are also in line with imaging findings demonstrating that the left and right ATL are linked via transcallosal ATL connections [74], hence forming a core system of semantics (see Figure 2). Progressive erosion of this bilateral system in neurodegenerative pathologies, occurring in left and right predominant forms of semantic dementia [75,76,77], inevitably leads to multidimensional semantic breakdown, including disorders of verbal semantics, visual-object semantics and social/emotional semantics. The weighting of such disorders depends on the left-right/right-left trajectory of the neurodegenerative process, which results in a graded time-dependent syndrome continuum incorporating phenotypical subdivisions related to the most impaired semantic dimension. Such subdivisions might include at least primary verbal-semantic impairments (currently referred to as ‘sv-PPA’; predominant left ATL damage), primary visual-semantic breakdown (‘progressive semantic agnosia/prosopagnosia’; predominant right ATL damage), primary social-semantic impairments (sometimes referred to as ‘semantic-behavioral variant FTD; predominant right ATL damage), as well as mixed phenotypes (often referred to as ‘semantic dementia’).

Yet, primary right ATL degeneration might raise questions given that it clinically leads to predominant behavioral disorders including compulsive, disinhibited and non-empathic behaviors, which are often more striking in every-day life than deficits of visual semantics [10,11,78]. Such behavioral disorders result from impairment of social/emotional semantics related to predominant right ATL damage, and probably to additional damage to behavior-controlling systems implemented by ATL-connected prefrontal systems comprising mesial-orbitofrontal regions (primarily damaged in behavioral variant FTD). Damage to ATL and prefrontal systems is coherent with neurodegenerative expansion in patients with predominant right ATL damage, and it also occurs, sooner or later, in patients with initial left ATL damage expanding to right ATL and prefrontal regions [75,77]. However, from a clinical/pragmatic point of view, categorizing predominant behavioral disorders as a particular phenotype of the semantic continuum might generate confusion among clinicians given its resemblance with behavioral variant FTD. Hence, several authors who characterized this clinical entity labelled it ‘right temporal variant FTD’ or ‘semantic-behavioral variant FTD’ [10,11] rather than considering it as a variant of a semantic spectrum disease.

An open issue of the graded semantic continuum, which is key to pharmacological approaches, is the potentially differential vulnerability to neuropathological processes of the left and right ATL, and more generally of distinct neurobiological areas within the ATL region. Neuropathological investigations suggest that both ATLs are mainly and similarly vulnerable to TDP-43 type C pathology [65], but other proteinopathies such as tauopathies have also been identified [33]. Hence, evidence for or against neurobiological subdivisions is rather weak mostly because of small sample sizes and the lack of in vivo biomarkers of proteinopathies. A second issue of clinical relevance is the incorporation of phenotypical subdivisions into the semantic disorder continuum to enable appropriate patient care, to adjust rehabilitation strategies, and to define target regions for therapeutical intervention. A last issue could potentially be the determination of diagnostic criteria and formal terminologies for impairments of the graded semantic continuum and its subdivisions with the aim to aid clinical reasoning, meaningful announcement to patients, and the construction of coherent research cohorts. The most integrative label might be ‘semantic dementia’, enriched by subdivisions differentiating verbal (‘sv-PPA’), visual, and social/behavioral variants. Work on this issue is currently supported by an Internal Working Group aiming at establishing a formal name and diagnosis criteria for the latter variant related to predominant right ATL damage [79].

### 4.3. Utility of the Graded Continua View for Clinical Practice and Research

**Figure 3:**
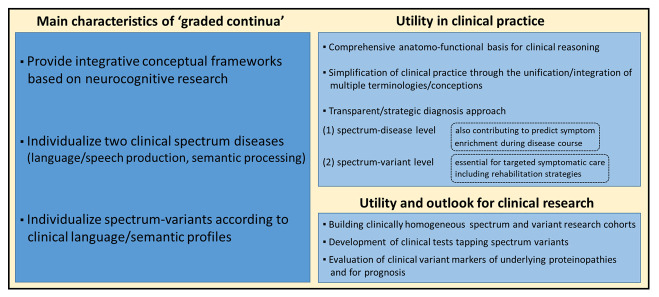
Main characteristics of graded continua and their utility for clinical practice and clinical research.

The conception of graded continua might be helpful for clinicians for several reasons (see Figure 3). First, ongoing debates about ‘splitting’ versus ‘lumping’ conceptions have generated confusion in the clinical community, and a comprehensive unifying framework could help clarify ideas while integrating multiple competing conceptions/terminologies such as ‘non-fluent PPA’, ‘agrammatic PPA’, ‘PPAOS’, ‘semantic PPA’, ‘semantic dementia’, ‘semantic-behavioral FTD’ or ‘right temporal variant FTD’. Second, the concept of continua provides a coherent and holistic view of two spectrum diseases related to degenerative erosion throughout two core systems of human cognition: the frontal language/speech production network (syntax–phonology–phonetics/AOS), and the bilateral ATL network of multidimensional semantics (Figure 2). Third, identifying subdivisions within the continua allows for refining phenotypical characterization, which might be key for clinical reasoning, meaningful announcement of the disorders to patients and caregivers (e.g., patients with predominant syntax deficits are distinct from patients with predominant articulation deficits), and for adjusting appropriate patient care. Graded continua also account for gradual symptom enrichment during the disease course and enable clinicians to clarify why and how deficits evolve throughout the language/speech production system and the semantic network (e.g., patients with failure of verbal semantics might progressively present deficits of recognizing the meaning of objects and faces, and of social interactions followed by behavioral disturbances).

The proposed frameworks also open avenues for future research while providing a consistent basis for the construction of coherent and homogeneous research cohorts (spectrum-variant cohorts, whole-spectrum cohorts). More particularly, they reinforce the need for developing internationally validated clinical tests individualizing and scoring AOS (vs. dysarthria), syntactic failure (vs. non-linguistic factors influencing sentence processing), and disorders of phonological encoding (vs. breakdown of phonological word representations). Similarly, despite the variety of available clinical tools for semantic disorders, there is still a lack of tests distinguishing deficits of verbal semantics from damage to lexical word-forms. Likewise, multidimensional semantic tools might allow for directly comparing verbal, non-verbal/visual semantics and social-emotional concepts. In keeping with this, a recent study has reported a multidimensional test (‘Fear and Spider Test’) implementing verbal, visual, auditory and emotional dimensions [80]. A second avenue for future research is to evaluate whether clinical subdivisions/variants of the two spectrum diseases might provide clinically relevant estimators of the underlying proteinopathy. Relatedly, underlying proteinopathies might provide clues for prognostic issues. Studies in PPA and FTD have indicated faster evolution when patients have autopsy-confirmed underlying Alzheimer pathology compared to patients with TDP-43 proteinopathies or tauopathies [81,82]. Similarly, there is evidence that nonfluent PPA, primarily caused by 4R-tauopathies, is faster evolving than semantic PPA, mainly caused by TDP-43 type C pathology [83].

### 4.4. Limitations

The proposal of ‘graded continua’ is based on a broad literature review using key words for research on PubMed, but it is not based on a systematic categorization of the identified research articles or any meta-analytic approach. It cannot be excluded that some published studies related to the topic have not been included or mentioned in this narrative review.

## 5. Conclusions

Neurology has undergone terminological and conceptual mutations in the field of PPA. This narrative review highlights that cognitive neuroscience and clinical research have shown that nfav-PPA and sv-PPA go beyond pure aphasia, and that they might be part of graded continua (spectrum diseases) with phenotypical subdivisions: syntactic/phonological/phonetic-AOS variants within the language/speech production continuum of the Broca-premotor/SMA network, and verbal/non-verbal/behavioral variants within the semantic continuum of the ATL system. The proposed holistic conceptual framework is thought to help overcome ‘lumping’ versus ‘splitting’ controversies by combining the notions of neurodegenerative continua and of clinically relevant graduations, which could be key for phenotypical characterization, symptomatic therapy and for building homogeneous research cohorts. Such a framework may entail subsequent development of operational criteria and formal terminologies for the two proposed spectrum diseases and their variant subdivisions. Future research will unveil whether variant graduations and linguistic/semantic features within the continua might be predictive of underlying proteinopathies and whether they might have prognostic value.

## Data Availability

No new data were created or analyzed in this study. Data sharing is not applicable to this article.
